# Complex transcriptional control of the AZFa gene *DDX3Y* in human testis

**DOI:** 10.1111/j.1365-2605.2010.01053.x

**Published:** 2011-02

**Authors:** M-A Rauschendorf, J Zimmer, R Hanstein, C Dickemann, P H Vogt

**Affiliations:** * Unit of Molecular Genetics & Infertility, Department of Gynecological Endocrinology & Reproductive Medicine, University of HeidelbergHeidelberg, Germany; † Department of Neuroscience, Albert Einstein College of Medicine, Kennedy CenterBronx, New York, USA

**Keywords:** AZFa gene expression, human Y chromosome, primates, spermatogenesis, transcriptional control

## Abstract

The human *DEAD-box Y* (*DBY*) RNA helicase (aka *DDX3Y*) gene is thought to be the major azoospermia factor a (AZFa) gene in proximal Yq11. Men with its deletion display no somatic pathologies, but suffer from complete absence of germ cells. Accordingly, DDX3Y protein is expressed only in the germline in spermatogonia, although the transcripts were found in many tissues. Here, we show the complex transcriptional control of a testis-specific *DDX3Y* transcript class with initiation at different sites upstream of the gene’s open reading frame (5′Untranslated Region; UTR) and with polyadenylation in their proximal 3′UTR. The most distal transcriptional start site (TSS; ∼1 kb upstream) was mapped in *MSY2*, a Y-specific minisatellite. As this testis-specific 5′UTR was subsequently processed by three alternative splicing events, it has been tentatively designated ‘exon-T’(estis). The *MSY2* sequence unit was also found upstream of the mouse *Ddx3y* gene. However, only after its tandem amplification on the Y chromosome of Platyrrhini (new world monkeys) and Catarrhini (old world monkeys) did *MSY2* become part of a novel distal promoter for *DDX3Y* expression in testis tissue and provides a second transcriptional start site (T-TSS-II) in Catarrhini. We therefore suggest that the development of a novel distal *DDX3Y* promoter in primates, which is activated only in testis tissue, is probably part of the gene’s germline translation control.

## Introduction

Large rearrangements on the sex chromosomes during mammalian evolution ∼200 million years ago (Mya) have led to distinct structures of the X and Y chromosomes in the human genome including the loss of many genes on the Y chromosome, which originally were present on the ancient sex chromosomes (Charlesworth & Charlesworth, 2000). It can therefore be assumed that the 16 presently still functional human X and homologous Y genes were evolutionarily selected for some male-specific functions (Lahn & Page, 1997; Skaletsky *et al.*, 2003). Examples are the anciently homologous X–Y gene pairs: *SOX3/SRY* (Marshall Graves, 2006), *TSPX/TSPY* (Delbridge *et al.*, 2004) and *RBMX/RBMY* (Elliott, 2004). According to the strata evolution model of Lahn & Page (1999), these X–Y genes belong to strata 1 and started diverging their genomic structures ∼200 Mya. Another functional homologous X–Y gene pair, diverged from regular meiotic recombination ∼80–130 Mya, is the human *DDX3X/DDX3Y* gene pair. It belongs to strata 3 (Lahn & Page, 1999).

*DDX3Y (aka DBY*; HGNC: 2699; GenBank accession no.: NM_004660) is one of the two Y genes mapped to the azoospermia factor a (AZFa) deletion interval in the proximal part of the long arm of the Y chromosome (Yq11.1; Vogt *et al.*, 1996; Lahn & Page, 1997; Vogt, 2005); its functional homologue on the X chromosome, *DDX3X* (aka *DBX*; HGNC: 2745; GenBank accession no.: NM_001356)*,* was mapped to the proximal part of the short X arm (Xp11.4; Park *et al.*, 1998). Although both genes lie outside the pseudoautosomal regions (PAR1 and PAR2) located at the tips of the sex chromosomes, they have a >94% sequence identity throughout the coding regions and their proteins are reported to be functionally exchangeable in the control of translation initiation in the cytoplasm (Chuang *et al.*, 1997) and of cell cycle progression at the G1-S phase (Fukumura *et al.*, 2003). Despite this functional equivalence and transcriptional activity of *DDX3Y* and *DDX3X* in each human tissue analysed (Lahn & Page, 1997; Ditton *et al.*, 2004), DDX3Y proteins were only found in pre-meiotic male germ cells (Ditton *et al.*, 2004). A germline-specific function of the *DDX3Y* gene was first indicated by absence of any somatic phenotype in men with AZFa deletions that also remove *DDX3Y* (Vogt *et al.*, 1996)*.* They all only suffer from severe testicular pathologies including the Sertoli-cell-only (SCO) syndrome (Vogt *et al.*, 1996, 2008; Foresta *et al.*, 1998; Kamp *et al.*, 2001; Krausz & Degl’Innocenti, 2006). It has been speculated that *DDX3Y* might be the major AZFa gene functionally required for male fertility because its deletion was found in men with the SCO syndrome (Foresta *et al.*, 2000). However, these gene deletions were not confirmed by sequence analyses and not yet found in other similar studies.

Deletion of the second AZFa gene, *ubiquitin-specific protease 9Y* (*USP9Y*; HGNC: 2633), was found to be compatible with normal sperm function (Luddi *et al.*, 2009), although probably with reduced fertility. Its phenotype looks heterogeneous, as in another case of a familial truncated *USP9Y* gene impaired spermatogenesis was reported (Krausz *et al.*, 2006). This suggests that the germline *USP9Y* expression, although not essential, might be able to ‘finetune’ the post-meiotic germ cell differentiation process in human. Indeed, USP9Y proteins were found to be expressed in spermatids (Vogt *et al.*, 2008).

Interestingly, truncation of the *USP9Y* gene was also found on the Y chromosome of fertile chimpanzee and bonobos (Tyler-Smith, 2008). Male fertility in these apes was obviously not hampered by loss of the *USP9Y* function. Instead, the *DDX3Y* gene is conserved on the Y chromosome of primates (Goto *et al.*, 2009) and the DDX3Y protein is expressed in the pre-meiotic germ cells (Ditton *et al.*, 2004). It can thus be assumed that *DDX3Y* is the major AZFa gene functionally required for human spermatogenesis (Vogt *et al.*, 2007, 2008; Tyler-Smith & Krausz, 2009).

In this article, we present experimental evidence for a large complexity of the human *DDX3Y* transcripts expressed in testis tissue. They initiate at different sites and the longest 5′UTR starts in a tandem repetitive sequence block known as the Y-specific ‘*MSY2* minisatellite’ (*DYS440*; Bao *et al.*, 2000). Processing for polyadenylation occurred at different sites in the proximal part of the ∼2.5-kb long 3′UTR. We found the basic *MSY2* sequence unit also upstream of the mouse *Ddx3y* gene. Tandem amplification on the Y chromosome was only found in the Platyrrhine (new world monkey) *Callithrix jacchus*, in the Catarrhine (old world monkey) *Macaca mulatta* and in the hominids (great apes) *Pongo pygmaeus* and *Pan troglodytes*. Testis-specific *DDX3Y* expression starting from these *MSY2* repeats was found in the catarrhine and the hominid primate class. We therefore like to propose that this primate-specific increase in the complexity of *DDX3Y* transcript variants expressed only in the male germline is part of a germline-specific translational control mechanism in human and most likely also in other primates, but not present in mouse.

## Materials and methods

Human blood and tissue samples used for DNA and RNA extractions were only collected by our collaborating clinical partners after medical indication and written consent of the patients. The study was approved by the local ethical commission of the University of Heidelberg. Primate tissue and blood samples were obtained from the German (DPZ) and Dutch (BPRC) primate centres via the EUPRIM network (EU contract: RII3-026155 of the 6th framework programme: http://www.euprim-net.eu). Before DNA and RNA extraction, they were only handled according to the current biosafety guidelines of the university.

### RNA isolation and RT-PCR analyses

RNA were isolated from all tissue samples with the Qiagen RNeasy Mini Kit (cat. no. 74106) using the manufacturer’s protocol (Qiagen GmbH, Hilden, Germany). Efficient on-column digestion of DNA during RNA purification was performed with the RNase-free DNAse digestion protocol (Qiagen, cat. no. 79254). For all reverse transcription-polymerase chain reaction (RT-PCR) assays, the first strand cDNA synthesis from the total RNA samples was performed after incubation with Oligo(dT)_15_ primer (Promega GmbH, Mannheim, Germany; cat. no. C1101) by RT with the Promega M-MLV reverse transcriptase enzyme (cat. no. M3683) following the manufacturer’s recommendations. About 1.3 μg of total RNA (producing ∼26 ng of cDNA from the polyA fraction) was used in each RT reaction. The quality of each cDNA sample was controlled by *β-actin* RT-PCR with species-specific oligonucleotides; its extension in the 5′UTR of the *DDX3Y* gene was with gene-specific oligonucleotides as described previously (Ditton *et al.*, 2004).

**Figure 2:**
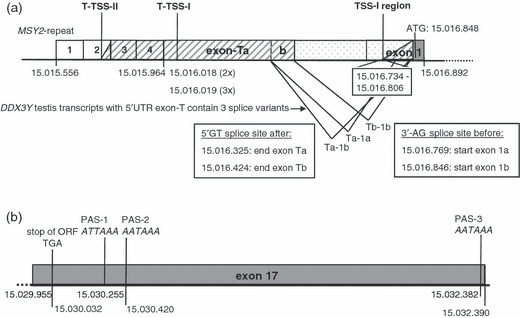
Schematic view on the genomic sequence structure of the *DDX3Y* gene in the current Y reference sequence (NC_000024; NCBI: build GRCh37) with the complexity of its putative transcriptional start sites (TSS) and polyadenylation sites (PAS) in the common and testis-specific transcripts. (a) 5′RACE and RT-PCR 5′UTR mapping experiments revealed a TSS for the common *DDX3Y* transcript class in exon 1 between positions 15.016.733 and 15.016.794 of the Y reference sequence (marked here as ‘TSS-I’ region). Two TSS were found for the testis-specific *DDX3Y* transcript class with long 5′UTR extensions defining ‘exon-T’. The first, tentatively designated ‘T-TSS-I’, was found by inspecting the sequence starts of five capped *DDX3Y* testis transcripts in the ‘database of TSS’ (http://dbtss.hgc.jp). They mapped downstream of *MSY2* in exon-T at the Y reference positions 15.016.018 and 15.016.019 respectively. The second, tentatively designated ‘T-TSS-II’, was mapped to the second repeat of the *MSY2* minisatellite (*MSY2-2*) located with four tandem repeats between Y sequence positions 15.015.556 and 15.015.964 respectively. Below the schematic *DDX3Y* exon-T-exon 1 structure, the three splicing variants of *DDX3Y* transcripts starting at ‘T-TSS-I’ or at ‘T-TSS-II’ in exon-T and designated as ‘Ta-1a’, ‘Ta-1b’ and ‘Tb-1b’, are tabled with their end and start positions after their 5′-GT and before their 3′-AG splice sites in exon-T and exon 1 respectively. (b) Schematic view of *DDX3Y* exon 17 with its three used PAS located between the TGA stop codon of the *DDX3Y* open reading frame at position 15.030.032 and the end of the 3′UTR at position15.032.390. Our 3′RACE experiments with RNA samples of multiple human tissues indicated that PAS1 and PAS2 were used only for testis transcripts and that PAS3 is the common site of *DDX3Y* transcripts expressed in all human tissues analysed.

All RT-PCRs were performed with the Invitrogen recombinant *Taq* DNA polymerase (cat. no. 10342-020; Invitrogen GmbH, Darmstadt, Germany) and using either 30 or 35 cycles with 1 μL of cDNA (∼1 ng) and optimal melting temperature (usually at 61 °C). The list of the used primer pairs is given in Tables S1 and S3 of the Supporting Information. They were designed from the genomic sequences given in the database to distinguish *DDX3Y* gene expression starting from different transcriptional start sites (‘TSS-I’ region; ‘T-TSS-I’ and ‘T-TSS-II’ respectively; see Fig. 2a). Primers were synthesized by Thermo Fisher Scientific (Ulm, Germany). For subsequent cloning and sequence analyses, we used the Promega pGEM-T easy vector system (cat. no. A1360) and the BigDye Terminator v1.1 Cycle Sequencing Kit (Applied Biosystems, cat. no. 4337450) with the ABI PRISM 3100 Genetic Analyzer (Applied Biosystems GmbH, Darmstadt, Germany).

### RACE experiments

All 5′- and 3′RACE experiments were performed with the RACE System of Invitrogen for Rapid Amplification of cDNA Ends (version 2.0; cat. no. 18374-058). In short, cDNAs were prepared as described before, but with gene-specific adaptor oligonucleotides containing two restriction sites for subsequent cloning and sequencing experiments. We used primers bridging *DDX3Y* exons 1–2 as reverse primers in 5′RACE experiments. In 3′RACE experiments, we used gene-specific adaptor oligonucleotides as forward primers upstream of the different 3′UTR polyadenylation sites (PAS) in *DDX3Y* exon 17 (PAS1, 2, 3; see Fig. 2b). To select subsequently for full-length 5′UTR cDNAs, the single-stranded 5′RACE cDNA products were tailed with dCTP and terminal deoxynucleotidyl transferase at their 3′ end. To select for cDNAs from the 3′UTR polyadenylated mRNA fraction, a oligo(dT) containing adaptor primer was used. In a second nested PCR experiment, an internal set of gene-specific primer pairs was used as reverse (5′RACE) and forward (3′RACE) primers respectively. PCRs were performed with the abridged universal amplification primer that was able to anneal selectively to the dC-tailed 5′ends of the 5′RACE cDNA products respectively, to the dT-tailed 3′ends of the 3′RACE products. Before sequence analyses, all RACE products were purified by cloning in the pCR2.1-TOPO vector (Invitrogen, cat. no. K4510-20). For preparative isolation of the recombinants, we used our standard lab protocols (Ditton *et al.*, 2004) for sequencing the BigDye Terminator v1.1 Cycle Sequencing Kit (Applied Biosystems, cat. no. 4337450) and the ABI PRISM 3100 Genetic Analyzer.

### RNA blot analyses

Two Normal multiple Human Adult Northern tissue blots (NmHAN-IIIA and NmHAN-VA; BioCat GmbH, Heidelberg, Germany) loaded with polyadenylated RNA pools (each 3 μg) from different human male and female tissues were hybridized with two different ^32^P-labelled PCR products generated from the 5′ end of a *DDX3Y* cDNA produced from transcripts of the exon-Ta-1b splicing variant. Both products were amplified with the same forward primer in 5′UTR exon-T (5′-TCA AGT CTG TCG AGC CTC TG-3′); starting at sequence position 54362 in the Bacterial Artificial Chromosome (BAC) RP11-475I1 sequence (GenBank accession no. AC004474) and one reverse primer bridging exons 2–3 (5′-CCT TTG CTC GCT GTA CTT GC’-3′) starting at sequence position 59572 in the same BAC sequence respectively, one reverse primer (5′-TTGACAAGCTTCAGCAAAGTTG-3′) starting in exon-T at sequence position 54621. RNA samples were run on denaturing 1% formaldehyde agarose gels and transferred to a charge-modified nylon membrane. Pre-hybridization and hybridization experiments were carried out with 5 mL of FastHyb-hybridization solution (cat. no. L1031250; BioCat GmbH, Heidelberg, Germany) according to the manufacturer’s instructions.

### ‘In silico’ analyses of nucleic acid sequences

Putative transcriptional start sites (TSS’s) of the murine *Ddx3y* and the human *DDX3Y* gene transcripts were analysed ‘in silico’ in the database of TSS (DBTSS; release 6.01) at the DBTSS web server (http://dbtss.hgc.jp) and compared with those listed in the GenBank database of the NCBI (http://www.ncbi-nih.gov) and ENSEMBL database (http://www.ensembl.org) respectively. Sequence comparisons and homology searches were performed by MegaBLAST and BLASTN via the BLAST server at NCBI. Multiple sequence alignments were performed using clustalW2, release 2.0.10, through the EMBL-EBI web server (http://www.ebi.ac.uk).

## Results

### Transcriptional start sites (TSS’s) in *DDX3Y* exon 1 produce variable 5′UTR lengths

We first explored the complexity of the testis-specific *DDX3Y* transcript population observed earlier on RNA blots (Ditton *et al.*, 2004) by 5′RACE experiments and sequence analyses of the isolated *DDX3Y* transcript lengths present in testis tissue and absent in leucocytes. The data are based on the genomic sequence information for the *DDX3Y* gene in the current Y reference sequence (NCBI database: NC_000024; GRCh37Y reference sequence assembly) with position of its ATG translational start codon at 15.016.848 Mb (Fig. S1).

We identified in the testis and leucocyte RNA samples, the putative commonly used *DDX3Y* TSS (referred to as ‘TSS-I’ from here onwards) upstream of the ATG codon in exon 1 between positions 15.016.744 and 15.016.794 in the Y reference sequence. We confirmed this result by additional RT-PCR assays with primer sets located around the proposed ‘TSS-I’ region. With cDNA samples from leucocytes and from different human tissues (testes, kidney, brain and spleen), the most distal forward primer with a positive RT-PCR signal in each RNA population was starting at Y sequence position 15.016.733; some further extension to position 15.016.627 was only observed for the testis cDNA sample (Table S1). We, therefore, assume a variable TSS of the common human *DDX3Y* transcript class defining the ‘TSS-I’ region between positions 15.016.733 and 15.016.794 of the Y reference sequence. The common 5′UTR length of *DDX3Y* exon 1 can thus be variable between 54 and 115 nucleotides (nt) respectively. In the testis tissue, it might extend further to 221 nt.

### Novel testis-specific *DDX3Y* transcripts with long 5′UTR extension start in *MSY2* repeats

With testis RNA samples, some clones of our 5′RACE experiments contained much longer 5′UTR sequences extending significantly beyond the commonly found 5′UTR exon 1 extensions. Sequence analyses found part of the tandemly repetitive *MSY2* sequence block in these testis transcripts. *MSY2* was described earlier as a human Y-specific minisatellite block with three, or more commonly, four tandem copies of a ∼100-nt sequence block containing a high sequence homology (*DYS440*; Bao *et al.*, 2000). As *MSY2* is located 885 nt upstream of the *DDX3Y* ATG translation codon (Fig. S1), our data suggest a significantly longer 5′UTR extension for these testis-specific *DDX3Y* transcripts most likely initiated from a novel distal *DDX3Y* promoter domain.

**Figure 1:**
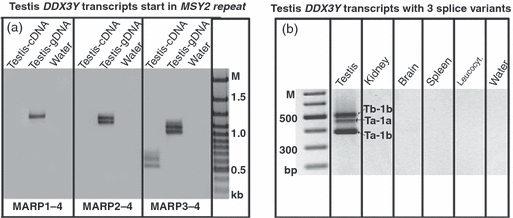
(a) Reverse transcription-polymerase chain reaction (RT-PCR) mapping of putative start site of the testis expressed *DDX3Y* transcripts with extended 5′UTR sequence in the *MSY2* repeat block. For all sequence information, see Table S1. To judge the specificity of the designed *MSY2* repeat-specific PCR assays, they were performed in parallel with cDNA samples of the polyadenylated testis mRNA fraction (testis-cDNA lanes) and with genomic DNA samples extracted from the same testis tissue [testis-g(enomic) DNA lanes; positive controls]. The water lanes control absence of any DNA and RNA contamination in the different PCR experiments (negative controls). We found two amplification products in the testis cDNA lanes only with the *MARP3-4* reaction, that is, when amplifying specifically the third (*MSY2-3*) and fourth (*MSY2-4*) repeats. This result maps the putative start site of *DDX3Y* testis transcripts into the second *MSY2* repeat (*MSY2-2*). Their different lengths compared with the genomic PCR products suggest some subsequent splicing processes. (b) Three different lengths of *DDX3Y* transcription products were identified in each RT-PCR assay independent of location of the primer sites along the upstream sequence of *DDX3Y* exon 1 and always only in the testis tissue, when performed with cDNA samples prepared from the mRNA pools of the kidney, brain and spleen and from leucocytes. Their sequence analyses identified three distinct splice variants as their common molecular origin (see Fig. 1). As no unspliced transcripts were found, this testis-specific 5′UTR was tentatively designated as ‘exon-T(estis)’ and its splicing variants coined ‘Ta-1a’, ‘Ta-1b’ and ‘Tb-1b’ respectively.

As clones containing tandem repetitive sequence blocks are well known to contain inherent instability, we wanted to confirm the transcriptional *DDX3Y start* region in *MSY2* by some additional RT-PCR assays with primer sets amplifying specific regions in the *MSY2* repeats (Table S1). By the parallel use of testis genomic DNA and cDNA as templates, amplification products including all *MSY2 repeats* (‘MARP1–4’ reaction) or the first and second *MSY2* repeats (‘MARP2–4’ reaction) were only found in the genomic PCR assays; the primer set amplifying specifically the third and fourth *MSY2* repeats (‘MARP3–4’ reaction) found two amplification products with a size difference of ∼100 nt also in the testis cDNA sample (Fig. 1a). Two cDNA amplification products indicated that the longest testis transcript should start upstream of the first site of the MARP3–4 reaction bridging the second and third repeats, thus in the second *MSY2* repeat (*MSY2–2*; Fig. S1). This was confirmed by subsequent sequence analysis; the additional 100 nt shorter RT-PCR product was produced by the second identical MARP3–4 site bridging the border of the third (*MSY2–3*) and fourth (*MSY2–4*) repeats. Both testis cDNA amplification products were significantly shorter than those of the corresponding genomic amplification products (Fig. 1a). It suggests that these transcripts were further processed by some splicing events.

To map and sequence the putative splice sites in the long 5′UTR sequence experimentally, we performed RT-PCR assays with a number of primer sets located along the upstream sequence of *DDX3Y* exon 1 until the *MSY2* repeats (Table S1). To study additionally the tissue distribution of these transcripts, we used polyA cDNA samples as templates, not only from leucocytes and from testis tissue but also from the kidney, brain and spleen tissues. Three distinct amplification products were found always, independent of location of the upstream primer site and always only with the cDNA sample of testis tissue (Fig. 1b).

Their sequence analyses revealed the use of two canonical splice donor (5′-GT) and two acceptor (AG-3′) sites in the amplified 5′UTR sequence parts (Fig. S1). We did not identify any unspliced long 5′UTR in these *DDX3Y* testis transcripts. It was therefore tentatively designated 5′UTR ‘exon-T’(estes) of the *DDX3Y* gene and its three alternative splicing products: ‘exon-Ta-1a’, -‘Ta-1b’ and -‘Tb-1b’ respectively (Fig. 1b).

Inspecting the ‘DBTSS’ (http://dbtss.hgc.jp) for *DDX3Y* testis transcripts with long 5′UTR extensions, we found five samples with part of the 5′UTR exon-T sequence. However, their TSS were not in the *MSY2* repeats, but always downstream of it, namely at the Y reference positions: 15.016.018 (two samples) and 15.016.019 (three samples) respectively (Fig. S1). They were found by using the RLM-RACE (RNA ligase-mediated rapid amplification of 5′ cDNA ends) protocol (Wakaguri *et al.*, 2008). As we expected that this and our 5′RACE protocol should deliver comparable results, we might have missed this alternative 5′UTR exon-T extension because we did not sequence all our 5′RACE amplification products, but only the longest ones. As independent from their start site, all *DDX3Y* transcripts with exon-T were expressed only in the testis tissue with the same three alternative splice variants (Fig. 1b), we can assume that this testis-specific *DDX3Y* transcript class has thus probably two distinct TSS in exon-T located in and downstream of the *MSY2* repeats. We tentatively designated them as ‘T-TSS-I’ and ‘T-TSS-II’ respectively (Fig. 2a).

### *MSY2* starting *DDX3Y*-exon-T testis transcripts have short 3′UTR lengths

On Northern blots, we and others observed polyadenylated *DDX3Y* testis transcripts with variable 3′UTR lengths. Besides an RNA population of ∼5 kb expressed in each tissue, the major testis RNA population had a length of ∼3 kb (Lahn & Page, 1997; Ditton *et al.*, 2004). This suggests their processing for polyadenylation in the proximal region of the usually ∼2.5-kb long 3′UTR sequence. We speculated that these testis-specific transcripts might also include those with the afore described 5′UTR exon-T extensions.

To explore this experimentally, two human tissue RNA blot experiments were performed with two *DDX3Y* cDNA probes both including exon-Ta downstream of ‘T-TSS-I’ (see Materials and methods). Both probes should therefore hybridize to all *DDX3Y* testis transcripts including exon-T, independent of their start at ‘T-TSS-I’ or at ‘T-TSS-II’ repectively.

**Figure 3:**
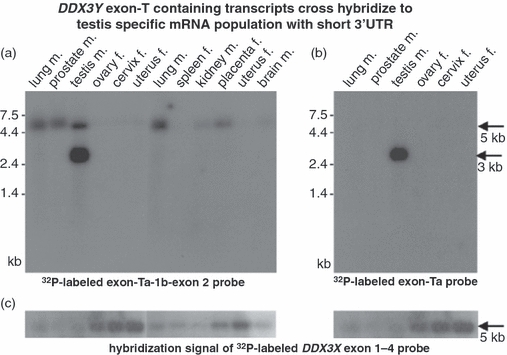
Two different ^32^P-labelled *DDX3Y* exon-T probes mainly cross-hybridized to the ∼3-kb long testis-specific mRNA population (marked by an arrow on the right). The Northern blots used in (a) and (b) contain the same quantities of polyadenylated RNA samples in each lane extracted from the tissues indicated (m, male tissue; f, female tissue). As the pure exon-Ta probe in (b) only cross-hybridized to the short testis-specific RNA population, the additional cross-hybridization of the exon-Ta-1b-exon-2 probe in (a) to the 5-kb long RNA class in different male tissues (marked by an arrow on the right) indicates that this additional hybridization signal is because of the *DDX3Y* exon-1b-exon 2 sequence part of this exon-Ta probe. The absence of cross-hybridization in RNA samples from female tissues confirms that both exon-T probes are only part of male-specific *DDX3Y* transcripts [the weak female placenta signal in (a) probably indicates some tissue contamination with embryonic male tissue]. (c) Cross-hybridization of a ^32^P-labelled *DDX3X* exon 1–4 probe to the ubiquitously expressed 5-kb long *DDX3X* transcripts (marked by an arrow on the right) is stronger in the female samples than in the male samples. This confirms the expression of *DDX3X* transcripts from both X chromosomes, and together with (a) and (b), assures the integrity of the RNA samples in each blot lane.

In both experiments, we found a strong hybridization signal with the short testis-specific RNA population (Fig. 3a,b). The additional weaker signal with the long transcript population (∼5 kb) in the different male tissues was only observed with the exon-Ta-1b probe including besides exon-Ta, the exon-1b part of exons 1 and 2 completely (Fig. 3a). This part of the probe is also present in all *DDX3Y* transcripts starting from the commonly used ‘TSS-I’ region of exon 1. The pure exon-Ta probe only cross-hybridized to the short transcript population of the testis tissue (Fig. 3b). Repeating the blot experiment with an exon-Ta probe located distal of ‘T-TSS-I’ and therefore identifying only those testis transcripts starting from ‘T-TSS-II’, we obtained the same result as shown in Fig. 3b. We conclude that all *DDX3Y* testis transcripts with the 5′UTR exon-T extension should become processed for polyadenylation in the proximal part of their 3′UTR in exon 17.

The proximal 3′UTR sequence of *DDX3Y* exon 17 in the Y reference sequence contains two possible internal PAS according to the rules of Sheets *et al.* (1990): one (PAS1) with a suboptimal consensus sequence (ATTAAA) and another (PAS2) with an optimal canonical ‘AATAAA’ sequence (Fig. 2b). Processing at both would fit to the calculated lengths of exon-T starting *DDX3Y* testis transcripts being in a range between 2.8 and 3 kb, depending on the exon-T splice variant included and their start at ‘T-TSS-I’ or at ‘T-TSS-II’ respectively (Fig. 2a,b). As PAS1 and PAS2 were separated by 165 nt in *DDX3Y* exon 17, some 3′RACE experiments were performed, which distinguished testis *DDX3Y* transcripts ending after PAS1 or PAS2 respectively (see Materials and methods). Sequence analyses of the cloned oligo(dT)-primed 3′RACE RT-PCR products revealed the use of both PAS, either PAS1 or PAS2, but only in the *DDX3Y* testis RNA population. In the RNA pools of leucocytes, *DDX3Y* transcripts ending behind PAS1 or PAS2 were absent and only transcripts containing the complete 3′UTR sequence, that is, using PAS3 (Fig. 2b), were identified.

Our 3′RACE assays thus confirmed that the short testis-specific *DDX3Y* transcripts are ending in proximal 3′UTR (Fig. 3a,b). Furthermore, we conclude that this transcript class mainly contains transcripts with two distinct long 5′UTR exon-T extensions (depending on their start at ‘T-TSS-I’ or ‘T-TSS-II’ respectively). This conclusion gained support from the complete sequence of an exon-T containing *DDX3Y* testis transcript published by the Mammalian Gene Collection (MGC) programme team (http://mgc.nci.nih.gov) in the GenBank database (accession no. BC034942). It starts in exon-T at the ‘T-TSS-I’ site and contains the major ‘Ta-1b’ splice variant. Its polyadenylation was found after PAS2 in the proximal 3′UTR.

### *MSY2* tandem repetition first occurs in primates

It has been reported that two tandem *MSY2* repeats (*MSY2-1* and *MSY2-2*) are also present upstream of the *DDX3Y* gene on the Y chromosome of the chimpanzee and orangutan (Bao *et al.*, 2000). We therefore wanted to know whether the testis-specific start site for *DDX3Y* transcripts in the *MSY2* repeats (T-TSS-II) is also present in these hominids (great apes) or only in human, the only species with three or more commonly four *MSY2* repeats (Bao *et al.*, 2000).

**Figure 4:**
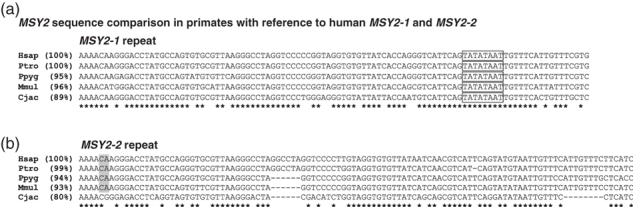
Sequence homology of the two *MSY2* repeats, *MSY2-1* (a) and *MSY2-2* (b), in different primate species. Alignments were performed with the clustalW2 program through the EMBL-EBI web server (http://www.ebi.ac.uk). Percentage data in brackets at the left indicate grade of sequence homology in the different primate species when compared with the human *MSY2*-1 and *MSY2*-2 sequences respectively. Stars underneath the aligned sequences indicate conserved nucleotides in all five primate species, dashes indicate missing nucleotides in comparison with another sequence because of insertion or deletion (indel) events. Sequence blocks boxed “TATATAAT” and grey toned (CA) are described in Discussion as putative conserved sequence elements of the proposed ‘core promoter’ in the *MSY2* repeats with ‘T-TSS-II’ location at the ‘CA’ dinucleotide of the *MSY2-2* repeat conserved only in the catarrhines.

In the database, genomic Y sequences upstream of and including the *DDX3Y* gene were found not only for the hominid, *P. troglodytes* in BAC clone CH251-128L22 (Genbank accession no. AC146254) but also for the catarrhine primate *M. mulatta* in BAC clone CH250-541A11 (GenBank accession no. AC213321) and for the platyrrhine primate *C. jacchus* in BAC clone CH259-161K20 (GenBank accession no. AC225609). Using these genomic BAC sequences, we found two *MSY2* tandem repeats located ∼1 kb upstream of *DDX3Y*, not only in the chimpanzee, as already suggested by Bao *et al.* (2000) but also in the other primates (Table S2). Most interestingly, comparative sequence alignment of the primates’*MSY2* repeats revealed a strong sequence homology to the first and second human *MSY2* sequence units in all catarrhine primates (>93%) and somewhat less (>80%) also in the platyrrhine species, *C. jacchus* (Fig. 4). When we screened the genomic Y sequence upstream of the mouse *Ddx3y* gene in BAC clone RP24-208N6 (GenBank accession no. AC145393) for *MSY2* homology, only one *MSY2*-like sequence stretch with highest homology to *MSY2-1* (54.5%) could be identified. Different deletion and insertion events on the mouse Y chromosome in this sequence region distinguished it from the same region conserved in primates significantly. This suggests that *MSY2* tandem repetition started and then became conserved after separation of the rodent and primate lineages, that is, ∼80–90 Mya.

### *MSY2* starting testis-specific *DDX3Y* transcripts with exon-T are only found in Catarrhini

Our comparative genomic Y sequence analyses upstream of the primates’*DDX3Y* gene identified not only conservation of the duplicated *MSY2* sequence unit but also downstream of it along the complete exon-T sequence including conservation of both 5′-GT splice donor sites and their corresponding 3′-AG splice acceptor sites in exon 1. We therefore speculated that the novel distal promoter domain of the *DDX3Y* gene used for the testis-specific initiation of *DDX3Y* transcripts with exon-T in human might have evolved already earlier during primate evolution.

To test this assumption experimentally, we extracted RNA samples from the testis, kidney, liver, spleen tissues and leucocytes of the primate species, *P. troglodytes*, *M. mulatta* and *C. jacchus*, the only primates for which currently these tissue samples were available via the EUPRIM network; http://www.euprim-net.eu). We analysed the putative 5′UTR extension of their *DDX3Y* transcripts along the homologous exon-T sequence by a series of RT-PCR assays that are able to distinguish different exon-T extensions (Table S3). As positive control, we also designed a primer set for analysing the start of the primates’ common *DDX3Y* transcript class expected to be in the same ‘TSS-I’ region as found for the human *DDX3Y* gene. A comparative RT-PCR analysis of transcripts from the mouse *Ddx3y* gene starting at ‘TSS-I’ or upstream of it was used as out-group.

When screening for transcripts with the ‘TSS-I’ homologous sequence region, amplification products of mouse *Ddx3y* transcripts were found in each cDNA sample and the same result was obtained for these *DDX3Y* transcripts in all primate species (Table S4). However, amplification of *DDX3Y* transcripts with 5′UTR extension in exon-T was only found in the testis RNA samples of the primates. In *C. jacchus*, it suggests a probable beginning of these transcripts in the ‘T-TSS-I’ homologous region downstream of *MSY2*; in the catarrhine *M. mulatta*, it suggests a probable transcription start in the ‘T-TSS-II’ homologous region in *MSY2*; similarly, this was found for the chimpanzee testis transcripts (Table S4).

**Figure 5:**
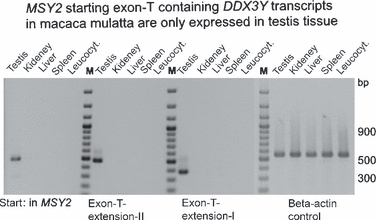
RT-PCR analyses of *DDX3Y* expression in different *Macaca mulatta* tissues and leucocytes (indicated above the gel slots) to analyse the putative extension of their homologous 5′UTR exon-T sequence in the corresponding transcripts. Similar quantities of the β-actin control in each RNA sample shown at the right confirm their general integrity. Three distinct primer sets called ‘in *MSY2*’, ‘exon-T-extension-I and -II’ respectively, were designed to distinguish the putative transcriptional start site (TSS) of *DDX3Y* transcripts along exon-T (for all sequence information, see Table S3). The first five lanes on the left will display only a positive reaction product if the transcripts would start in *MSY2* at a ‘T-TSS-II’ homologous site. The following second five lanes would also display a positive reaction product if the transcripts would start downstream of *MSY2* but upstream of ‘T-TSS-I’. The third five lanes would only display a positive reaction product if the transcripts would start downstream of this ‘T-TSS-I’. All cDNAs were obtained from RNA pools extracted from the same fertile male rhesus monkey. The DNA marker given at the right is a 100 bp DNA ladder [GeneRuler 100 bp DNA Ladder Plus; Fermentas; Fermentas GmbH, St. Leon-Rot, Germany]. RT-PCR amplification products were always only identified in the cDNA samples of the testis tissue. Its putative start site in *MSY2* (indicated by amplification product in the testis lane on the left) suggests that the longest testis-specific *DDX3Y* transcripts would start in a similar location of the *MSY2* repeats of *M. mulatta* as found in human.

Moreover, as shown in Fig. 5 for the samples of the macaque, the lengths of the primates’ testis-specific exon-T amplification products suggest that these transcripts are spliced like the human exon-T containing *DDX3Y* transcripts (Fig. 5). We therefore sequenced these testis-specific RT-PCR products with exon-T extension from all primates; they always displayed the same splice junction sites as found for the major human exon-Ta-1b variant. No indication was found for one of the other human exon-T splice variants (‘Ta-1a’ and ‘Tb-1b’) in the primates’ testis transcripts. The same exon-Ta-1b splice variant was also found for the shorter 5′UTR exon-T sequence of the *C. jacchus DDX3Y* testis transcripts. Formation of 5′UTR exon-T by this splicing event seems therefore to be conserved in different primate lineages and independent from the exon-T start site, being in a homologous ‘T-TSS-I’ or ‘T-TSS-II’ region respectively.

In summary, we propose that duplication of the *MSY2* sequence unit in primates might have triggered the development of a novel distal promoter domain for the *DDX3Y* gene that is able to initiate a novel testis-specific transcript class with longer 5′UTR extensions and the formation of exon-T by a subsequent splicing process. Most likely, after separation of the Platyrrhini and Catarrhini primate lineages, that is, ∼38 Mya (Goodman *et al.*, 2005), only the catarrhine primate lineage then evolved a second TSS (T-TSS-II) in the duplicated *MSY2* sequence block providing the same transcript class with still longer 5′UTR extensions. Besides ‘Ta-1b’, the alternative exon-T splice variants, ‘Ta-1a’ and ‘Tb-1b’, were evolved only recently after divergence of the human and chimpanzee lineages, that is, 6–7 Mya.

## Discussion

### Novel male germline-specific *DDX3Y* transcription products in primates

Most genes functionally expressed only during spermatogenesis are known to be under the pressure of sexual selection to maximize reproductive success (Kleene, 2003). This often leads to complex testis-specific patterns of their transcription products which help control their translation in the different testicular cell types. We believe that one example of such a strong gender-related gene in human spermatogenesis is probably the *DDX3Y* gene. The data presented in this article point to evolution of a novel distal promoter domain for the *DDX3Y* gene on the Y chromosome of primates, which is not present on that of the mouse upstream of the *Ddx3y* gene. Most interestingly, this second *DDX3Y* promoter domain seems to become activated only in the male germline and initiates a complex pattern of transcription products with distinct 5′UTR extensions processed at different splice-junction sites and polyadenylated in the gene’s proximal 3′UTR.

In mouse, *Ddx3y* is expressed in all tissues analysed; two transcript variants were found distinguished by different lengths of their 3′UTR (Vong *et al.*, 2006). In contrast to human, there is no evidence for requirement of the Ddx3y protein at any stage of mouse spermatogenesis (Mazeyrat *et al.*, 2001). It is believed that this function is fulfilled here by an autosomal retrogene of the homologous *Ddx3x* gene (*PL10/D1Pas1* located on chromosome 1), which is expressed only in the testis tissue (Leroy *et al.*, 1989; Session *et al.*, 2001). In human, the *RBMY* gene located on the long Y arm in the AZFb deletion interval seems to have followed a similar evolutionary pathway (Elliott *et al.*, 2000). It has a functional X homologue (*RBMX*) and an autosomal retrogene on chromosome 11 (*HNRNPG-T*) expressed in the nuclei of meiotic spermatocytes. However, a functional autosomal *DDX3Y* (or *DDX3X*) retrogene cannot be found. The two autosomal *DDX3X* homologous genes on chromosomes 4 and 20 contained truncated protein coding frames defining them as non-functional pseudogenes (Kim *et al.*, 2001).

Instead of this, the genomic Y structure upstream of the primates *DDX3Y* gene was found to be conserved and developed a novel promoter domain with functional restriction to the male germline. As in human, the non-human primates also express *DDX3Y* transcripts in the testis tissue, which contains the exon-T 5′UTR (Table S4). Its different extensions in the platyrrhine and catarrhine primate lineages suggest that development of a second start site for these transcripts in the *MSY2* repeat (T-TSS-II) was probably a following and independent second evolutionary step in this genomic Y region aimed at further optimizing the *DDX3Y* expression profile in the catarrhines’ testis tissue. We are not yet aware whether both TSS were indeed used in these catarrhines like in human. Nor are we aware of the cellular distribution of the DDX3Y protein in these primates. However, the evolutionary conservation of the novel distal promoter domain upstream of the *DDX3Y* gene in different primate lineages, always activated only in testis tissue, led us to speculate that its protein expression in these primates might be restricted to the male germline as in human as well. If this holds true, the distinct 5′UTR extensions of their *DDX3Y* testis transcripts have developed during primate evolution as an essential part of the gene’s germline-specific translation control.

### Is *MSY2* tandem duplication involved in development of the germline-specific distal promoter domain for the primates’*DDX3Y* gene?

Short tandem repetitive sequence units like *MSY2*– also called minisatellites – are well known in the literature as functional enhancer elements for the transcriptional activation of upstream or downstream protein coding genes (e.g. Paquette *et al.*, 1998 and references therein). However, these regions are also often prone to genomic instability and cause hotspots for meiotic recombination (Jeffreys *et al.*, 1998). Consequently, minisatellite sequence units are usually not conserved beyond closely related species unless their sequence variation is functionally restricted. We can assume that the high sequence conservation, which we found for the *MSY2* repeats in primates (Fig. 4), suggests some functional constraints on the complete *MSY2* sequence structure. Probably, the already duplicated *MSY2* sequence unit on the *C. jacchus* Y chromosome has evolved some sequence features, which supported the development of a novel distal promoter region for *DDX3Y* on this primate Y chromosome. These *MSY2* repeats might then have served as functional important enhancer domains to control its activation only in the testis tissue.

Short tandem repeats contain an inherent ‘enhancer’ quality to control the transcriptional efficiency of the downstream gene (Lee *et al.*, 2008). Multiple examples are therefore known where variation in sequence or copy number of a minisatellite located upstream of a gene can cause significant human pathologies (e.g. insulin-dependent diabetes mellitus type 2 or IDMM2; Kennedy *et al.*, 1995). We have not yet found any variation in sequence and copy number of the *MSY2* repeats in the DNA samples of our European patient collective with idiopathic azoospermia. We also did not find any men with only three *MSY2* tandem repeat*s* as described by Bao *et al.* (2000) in a fertile male population in southern China. Probably, there is a functional need for the complete *MSY2* structure in most human populations, which only allows a restricted copy variation without causing some germline pathology. Based on these data, it is therefore most likely that the tandem repetitive structure of *MSY2* is indeed a functional part of the novel distal promoter domain of the primates’*DDX3Y* gene.

### There are two distinct testis-specific TSS in human 5′UTR exon-T

Further *MSY2* sequence evolution in the Catarrhini lineage has developed a second start site for the testis-specific *DDX3Y* transcript class in its repetitive sequence structure. In human, the result is six testis specific variants because the two distinct exon-T extensions each are processed with three alternative splice variants, ‘Ta-1b’, ‘Ta-1a’, and ‘Tb-1b’ respectively (Fig. 2a).

Surprisingly, we did not find any 5′-capped *DDX3Y* human testis transcripts starting with *MSY2-2* in the ‘DBTSS’ (http://dbtss.hgc.jp; Wakaguri *et al.*, 2008). Probably, isolating stable minisatellite sequence repeats in the used cloning vectors was not possible. Alternatively, lack of these transcripts in the database would indicate that *DDX3Y* testis transcripts starting with *MSY2-2* might not be capped during their further processing.

Our RT-PCR assays with different primer sets along exon-T (Fig. S1b & Table S1) revealed that independent of these start sites, all exon-T containing *DDX3Y* transcripts are expressed as testis-specific and all are spliced at the same splice-junction sites. We therefore concluded that they belong to the same testis-specific *DDX3Y* transcript class, which starts downstream of *MSY2* at ‘T-TSS-I’, or at ‘T-TSS-II’ in the second *MSY2* repeat respectively. Unfortunately, we did not succeed in mapping the precise location of ‘T-TSS-II’ in the *MSY2-2* repeat experimentally because cloning of the corresponding capped 5′UTR sequence by the RLM protocol of Wakaguri *et al.* (2008) was also not possible.

Extensive analyses for conserved sequence motifs in putative TSS of eukaryotic genes have revealed a ‘YA’ dincucleotide (nucleotide code is according to the IUPAC table given at: http://www.bioinformatics.org/sms/iupac.html; TSS nucleotide marked underlined) as the most important ‘TSS’ sequence element conserved from yeast to man (Smale & Kadonaga, 2003). It is part of the so-called ‘Inr’ motif (consensus: YYANWYY) located – if functional – within a small range downstream of a second conserved sequence motif called the ‘TATA box’ (consensus: TATAWAAR). Both sequence motifs, when spaced in a functional distance to each other (28–34 nts; Ponjavic *et al.,* 2006), have been also coined ‘core promoter’ because this context is sufficient to bind efficiently the general transcription factors, such as the TFIID complex (Juven-Gershon *et al.*, 2008).

When we screened the conserved *MSY2* repeats for such sequence motifs, we found a nearly perfect TATA box in the *MSY2-1* repeat of all primates linked to a putative initiation start motif (CA) in the *MSY2-2* repeat within an optimal distance (30 nt) only in the catarrhines and not in *C. jacchus* (Fig. 4). Although this *‘*in silico’ analysis for a putative ‘core promoter’ structure in *MSY2* would nicely confirm the experimental outcome of our RT-PCR assays predicting ‘T-TSS-II’ to be located in *MSY2-2*, we cannot rule out some variability in the proposed ‘T-TSS-II’ site in these repeats because of the presence of more conserved ‘YA’ dinucleotides within other putative ‘Inr’ motifs in the *MSY2* repeats.

### Post-transcriptional control of the testis-specific *DDX3Y* transcript class by alternative exon-T splicing and 3′UTR PAS

In all non-human primates analysed, the testis-specific *DDX3Y* transcripts with exon-T were further processed by a specific splicing event designated as ‘Ta-1b’ according to the human major splice variant having the same splice-junction sites. RT-PCR assays, which analyse the 3′UTR extension of these primates’ exon-T transcripts, suggested similar PAS in their proximal 3′UTR as we found for the use of human exon-T transcripts (data not shown).

Post-transcriptional alternative splicing events, with or without alternative 3′UTR polyadenylation processes, are found for ubiquitously transcribed genes with functional expression of some of their transcript variants at distinct phase(s) of the male germline (Elliott, 2003; Kleene, 2003; Ehrmann & Elliott, 2005). To ensure their tissue-specific splicing processes, they usually require testis-specific cofactors binding together with the common spliceosome protein complex to the specific target sites. Similarly, to initiate the recognition of testis-specific PAS in the proximal 3′UTR, tissue-specific protein variants are required, which bind to the common 3′UTR cleavage protein complex, thereby directing the binding to specific 3′UTR sequence motifs.

In human and mouse, prominent examples of such testis splicing cofactors are two members of the RNA Binding Motif (RBM) family, RBMY and hnRNPGT (Elliott, 2004). Interestingly, the RBMY protein is encoded by a Y gene on the mouse and human Y chromosome and hnRNPGT by its autosomal retrogene in mouse and human as well. Functional assays for analysis of testis-specific 3′UTR-binding proteins involved in 3′UTR processing with subsequent polyadenylation were performed in the mouse. Most common is probably a variant of the cleavage stimulation factor (CstF)-64, the RNA-binding component of the general CstF binding downstream of the mRNA cleavage site. It is encoded on the X chromosome and has an autosomal paralogue, τCstF-64 (gene name *Cstf2t*), which is expressed only during meiosis and post-meiotic germ cell development (Dass *et al.*, 2007). The 3′UTR cleavage factor-I (CFIm) also supports the polyadenylation process in the proximal 3′UTR of mouse transcripts expressed during spermatogenesis, most interestingly, with a preference for non-canonical PAS as found here for the testis-specific PAS1 site (Sartini *et al.*, 2008).

It has been also repeatedly argued that there might be some functional interactions between distinct 5′ and 3′ termini of the polyadenylated mRNAs for controlling their translation in distinct cells differentially (Gallie, 1998; Kozak, 2004; Hughes, 2006); and especially in male germ cells (DeJong, 2006; Kolthur-Seetharam *et al.*, 2008). It is therefore most likely that the complex transcriptional control, which we have described here for the *DDX3Y* gene in human testis tissue, is indeed an essential part of its observed strict translation control mechanism allowing significant expression of the DDX3Y protein only in the pre-meiotic germ cells (Ditton *et al.*, 2004). Further functional analyses of these testis-specific *DDX3Y* transcript variants will now have to reveal the molecular tools of this germline-specific translation programme.

## Supporting Information

**Figure S1.** Genomic sequence region of the human Y chromosome reference in the NCBI database (accession no. NC_000024; GRCh37) starting from position 15.014.702 upstream of the *MSY2* sequence block to position 15.016.892, the end of *DDX3Y* exon 1.

**Table S1.** List of oligonucleotides used as primer sets for the three RT-PCR assays described in this article.

**Table S2.** Sequence positions and extensions of *MSY2-1* and *MSY2-2* repeats in the genomic BAC clones of the primates.

**Table S3.** List of the species-specific oligonucleotides designed from genomic BAC sequences containing the homologous 5′UTR exon-T sequence upstream of the human and primates' *DDX3Y* gene.

**Table S4.** Outcome of the comparative exon-T expression assays in tissues and leucocytes of some primates and human and of mouse as out-group.

As a service to our authors and readers, this journal provides supporting information supplied by the authors. Such materials are peer-reviewed and may be re-organized for online delivery, but are not copy-edited or typeset. Technical support issues arising from supporting information (other than missing files) should be addressed to the authors.
