# Supplementary material for: Complex transcriptional control of the AZFa gene DDX3Y in human testis
Source: Int J Androl. 2011 Feb;34(1):84–96. doi: 10.1111/j.1365-2605.2010.01053.x (PMC3039753; doi:10.1111/j.1365-2605.2010.01053.x)
Supplement: Supplementary file 1 [file ijan0034-0084-SD1.pdf]

Fig. 1 supporting information Rauschendorf et al.

Genomic sequence region of the human Y chromosome reference in the NCBI database (acc. no. NC_000024; GRCh37) starting from position 15.014.702 upstream of the *MSY2* sequence block to position 15.016.892, the end of *DDX3Y* exon 1.

15.014.702 cagtgtagcagtggcggtcatttacatgccaaaatacatattttattataaatattcttt

15.014.762 taattatataataattaggtttgttaggggccagaggggtgtcattgtgcatcatttgag

15.014.822 tttatttctttgggaggcaaagagagaggaaaggaaggtcaaaaatggagaaggccaata

15.014.882 aatttattttatatcacgtgtgaatttaacttgagaatggtaaaggtgtcgacacaaaat

15.014.882 atgttataaaaaaggaaatctagttgacaactttaacttgccaaagcagtaaaccaactt

15.015.002 cactctgggaacactgaatagaaagtgtatgttccaatcactttgaatgttttaattta

15.015.062 aattcggaaaagcagaaatatttgtgataatgttttgagaatggaggtatttgaattta

15.015.122 gcactaggaaaccctttgaaggacggtgtaaaatctggatatttaatctggtttcgctga

15.015.182 gagttcagagggaagaagtgtttgtaaaaccacgtatatggctattagttaaagccagga

15.015.242 atctagtactaagctttttttttttttttttttttttcctgcctgtctgctgaatctgg

15.015.302 actagagggaggactgagaatttggccaaaaaaagtaactccttctagggcaaaagacta

15.015.362 tttcccacccactgcagcgcctgtaagttgttacgaacgtaggacaactttggtgtagtg

15.015.422 gttccttcagtaggccgatccccctcaaccctcattcttttgggcaaccaaaagagtctt

15.015.482 tcacaatcccatggctaggctggctttggggacgtagtatagcactggttaacctaggtt

15.015.542 gcagggtgcccaga | **aaaacaagggacctatgccagtgtgcgttaagggcctaggtccccc** ***MSY2***

15.015.602 **ggtaggtgtgttatcaccagggtcattcagtatataattgtttcattgtttcgtg | aaaac**

15.015.662 **aagggacctatgccagggtgcgttaagggcctaggcctaggtccccttgtaggtgtgtta**

15.015.722 **taatcaacgtcattcagtatgtaattgtttcattgtttcttcatc | actatgccagggtgc**

15.015.782 **gttaagggcctaggcctaggtcccctggtaggtgtgttatcatcaacgtcattcagtatg**

15.015.842 **taattgtttcattgtttcttcatc | actatgccagggtgcgttaagggcctaggcctaggt**

15.015.902_**cccctggtaggtgtgttatcatcaacgtcattcagtatgtaattgtttcattgtttcttc**

15.015.962 **atcc** | tgtgatgctaaagccgtatgc*tcataaa*gcgcaggcacaccggtagaaaaac**ta**ga

15.016.022 ggggtctggagttc**c**taggtgaaccccagcagcctaaccctgtcaagtctgtcgagcctc

15.016.082 tggctggtcacacgtgctgcggaatccactcagctttcctcaggtgcagtcaggtccatc

15.016.142 ctgcagagggaccttctgcggacctgttctttcacctccctaacctgaagattgtattca

15.016.202 aaccaccgtggatcgctcacgtaaaatggtcactgcgcctaacacctgggatcccgtaac

15.016.262 ccttatctatcttggcttcagagagtttttttgactagttccaactttgctgaagcttgtc

end of exon-Ta at 5´-GTsplice site

15.016.322 aaag**GT**aggtgacggctagttggaacggaaaaattttacgaaacttcctattctcagaag

end of exon-Tb at 5´-GTsplice site

15.016.382 taaaagggaagagagagtgcttaaggaagaagggaagttgagg**GT**gggtaaggagggagc

15.016.442 gggagttagtggtagattgtcactgtgtttaagatttccccaaggcgaaaaaggcgaaag

15.016.502 atatcttgctagatccctagaattcgaaggcattaggagagggcggggatagcaaacatc

15.016.562 gcgcgaattttgagaggcgctgggactacgtaatcccgcgatcttatgactaaacgaacg

15.016.622 cgatttagggagaagtaacggtagcgataaagggattgggtggtggtggtggaggggtgg

15.016.682 agccaacgattacgcacgttgtgcgtgctgtcgtatagctgtgggaggggccatattacc

exon-1a after 3´-AG splice site

15.016.742 gcgtaggctaaccagtgcgcttaat**AG**ctgtaggtccagtgtaagagttccgctattcgg

exon-1b after 3´-AG splice site

15.016.802 tctcacacctacagtggactacccgattttcgcttctcttc**AG**gg***ATG***AGTCATGTGGT

15.016.862 GGTGAAAAATGACCCTGAACTGGACCAGCAG 15016892 **end of *DDX3Y* exon 1**

Sequence of *MSY2* repeats 1 to 4 (*MSY2-1* to *MSY2-4*) separated by "|") is given with bold type letters. Location of the different MARP primer sequences listed in Table 1 of the supporting information (MARP1 to MARP4) are underlined in the corresponding Y reference positions. They were used for mapping the putative start of the testis specific *DDX3Y* testis transcript class in the second *MSY2* repeat by RT-PCR (Fig. 3a). Another transcriptional start site for five *DDX3Y* testis transcripts of the same class was mapped by the DBTSS database (http://dbtss.hgc.jp) downstream of *MSY2* at sequence position 15-016.018-15.016.019 (the corresponding dinucleotide "ta" is underlined and marked bold type).

The two different 5´-GT- splice donor and 3'-AG- splice acceptor sites given in boxed bold capital letters were identified by sequence analyses of the three exon-T splice variants, "Ta-1a"; "Ta-1b"; "Tb-1b", as described in the main text. They mark the extension of exon-Ta and exon-Tb, respectively, the starts of exon-1a and exon-1b which are fused alternatively depending on the exon-T exon-1 splice event. The translational start codon "ATG" of the *DDX3Y* gene in exon 1 is marked bold type and italic. Begin of the *DDX3Y* open reading frame is given with capital letter code.
