# Supplementary material for: Complex transcriptional control of the AZFa gene DDX3Y in human testis
Source: Int J Androl. 2011 Feb;34(1):84–96. doi: 10.1111/j.1365-2605.2010.01053.x (PMC3039753; doi:10.1111/j.1365-2605.2010.01053.x)
Supplement: Supplementary file 2 [file ijan0034-0084-SD2.pdf]

Table 1 supporting information Rauschendorf et al.

List of oligonucleotides used as primer sets for the three RT-PCR assays described in this paper.

The list is subdivided according to the three PCR assays which we designed for analysis of the distinct proximal and distal 5´UTR sequence extensions of the human *DDX3Y* transcripts. All forward primers (marked by appendix “for”) were designed in and upstream of their 5´UTR sequences proposed by the 5´RACE experiments. The common reverse primer (marked by appendix “rev”) in each PCR assay is bridging exon-2-3 to ensure the amplification of only the cDNAs of *DDX3Y* transcription products. Its location is given by its end position in exon-3. Besides the start positions of all forward primers in the BAC clone sequence, their positions in the current Y reference sequence (NC_000024; GRCh37) are given as well. In the last column, presence (+) of the amplification product indicates that the forward primer used is proximal to the TSS analysed; absence (-) of the amplification product indicates that the forward primer used is distal of the TSS and therefore not present in the 5´UTR sequence of the analysed *DDX3Y* transcript.

**Common TSS-I 5´UTR extension assay**	**primer sequence**	**BAC RP11-475I1 seq. start position**	**Y reference NC_000024 seq, start position**	**RT-PCR assay “+” = present; “-“ = absent**
1261for	TCATGTGGTGGTGAAAAATGAC	55150	15.016.853	+
2067for	CATATTACCGCGTAGGCTAA	55031	15.016.733	+
2405for*	TAGGGAGAAGTAACGGTAGC	54925	15.016.627	-
2389for	CTACGTAATCCCGCGATCTTATG	54885	15.016.587	-
reverse primer				
1286rev		59572	15.021.274	

* this RT-PCR reaction was only positive for the testis RNA sample

***MSY*-repeat start assay**	**primer sequence**	**BAC RP11-475I1 seq. start position**	**Y reference NC_000024 seq. start position**	**RT-PCR assay “+” = present; “-“ = absent**
MARP1for	CTGGCTTTGGGGACGTAGTA	53798	15.015.501	-
MARP2for	AAAACAAGGGACCTATGCCAG	53854 + 53955	15.015.556<br>15.015.657	-
MARP3for	TCACTATGCCAGGGTGCGTTAA	54063 + 54162	15.015.765<br>15.015.864	+
reverse primer				
MARP4rev		55187	15.016.889	

***MSY2*- 5´UTR proximal extension assay**	**primer sequence**	**BAC RP11-475I1 seq. start position**	**Y reference NC_000024 seq. start position**	**RT-PCR assay “+” = present; “-“ = absent**
1726for	CTGTGATGCTAAAGCCGTATGC	54263	15.015.965	+
1313for	TCAAGTCTGTCGAGCCTCTG	54362	15.016.064	+
1394for	CGTAACCCTTATCTATCTTGG	54554	15.016.256	+
2389for	CTACGTAATCCCGCGATCTTATG	54885	15.016.587	-
reverse primer				
1286rev	CCTTTGCTCGCTGTACTTGC	59572	15.021.274	
