# Supplementary material for: Complex transcriptional control of the AZFa gene DDX3Y in human testis
Source: Int J Androl. 2011 Feb;34(1):84–96. doi: 10.1111/j.1365-2605.2010.01053.x (PMC3039753; doi:10.1111/j.1365-2605.2010.01053.x)
Supplement: Supplementary file 3 [file ijan0034-0084-SD3.pdf]

Table 2 supporting information **Rauschendorf et al.**

Sequence positions and extensions of *MSY2-1* and *MSY2-2* repeat in the genomic BAC clones of the primates here listed by their species code given in the first column. Abbreviations used are: Hsap = Homo sapiens, Ptro = Pan troglodytes, Ppyg = Pongo pygmaeus, Mmul = Macaca mulatta, Cjac = Callithrix jacchus. No genomic BAC clone sequence is available for Pongo pygmaeus. This genomic sequence was isolated by PCR and deposited in the GenBank under the given accession number (Bao et al., 2000). The BAC clones identified are listed by their BAC-library and number code and GenBank accession number; The *MSY2* sequence polarity in the genomic clones is given by "+"= 5´-3´ and "-"= 3´- 5´.

**primate species**	**BAC library and number code**	**GenBank accession no.**	***MSY2*-seq. polarity.**	***MSY2-1* position**	***MSY2-2* position**
Hsap	RP11-475I1	AC004474	+	53854 – 53954	53955 – 54064
Ptro	CH251-128L22	AC146254	-	60116 – 60016	60015 – 59907
Ppyg	---------------------	AF207846	+	36 – 136	137 – 240
Mmul	CH250-541A11	AC213321	-	84085 – 83985	83984 – 83881
Cjac	CH259-161K20	AC225609	-	52656 – 52556	52555 – 52460
