# Supplementary material for: Complex transcriptional control of the AZFa gene DDX3Y in human testis
Source: Int J Androl. 2011 Feb;34(1):84–96. doi: 10.1111/j.1365-2605.2010.01053.x (PMC3039753; doi:10.1111/j.1365-2605.2010.01053.x)
Supplement: Supplementary file 4 [file ijan0034-0084-SD4.pdf]

Table 3 supporting information   Rauschendorf et al.

List of the species-specific oligonucleotides designed from genomic BAC sequences containing the homologous 5´UTR exon-T sequence upstream of the human and primates´ *DDX3Y* gene (a genomic Y reference sequence is not yet published for the non-human primate species). The species-specific RT-PCR assays in the different primate tissues were performed with primer sets able to distinguish *DDX3Y* transcripts starting from the in human defined common "TSS-I region" or starting from the human "T-TSS-I" homologous region in exon-T (5´UTR exon-T extension-I), respectively, from the human "T-TSS-II" homologous region in *MSY2*. To distinguish putative 5´UTR exon-T extensions downstream of *MSY2* but extending beyond the putative "T-TSS-I" region, an additional primer set was designed in the homologous exon-T regions for a second exon-T RT-PCR assay, marked "5´UTR extension-II". Forward primers located at similar locations in the *MSY2* containing upstream region of the mouse *Ddx3y* gene were designed to include a comparative expression analysis for the mouse gene as an out group. The used homologous reverse primers always bridge the *DDX3Y* exon 2-3 sequence blocks.

RT-PCR assay for:	species-specific primer seq.:	BAC code (with strand polarity):	BAC position:
**TSS-I:**			
Homo sapiens (Hsap)	CATATTACCGCGTAGGCTAA	RP11-475I1 (plus strand)	55031
Pan troglodytes (Ptro)	CATATTACCGCGTAGGTTAAGC	CH251-128L22 (minus strand)	59139
Macaca mulatta (Mmul)	CATATTACCGCGTAGGCTAA	CH250-541A11 (minus strand)	83107
Callithrix jacchus (Cjac)	GCCATTTTAAGACGGAGTCTAAG	CH259-161K20 (minus strand)	51750
Mus musculus (Mmus)	ACTCGTCAGTACGTTTGGG	RP24-208N6 (minus strand)	93858
**T-TSS-I<br>5´UTR exon-T extension-I:**			
Homo sapiens (Hsap)	TCAAGTCTGTCGAGCCTCTG	RP11-475I1 (plus strand)	54362
Pan troglodytes (Ptro)	TCAAGCCTGTCGAGCCTCTG	CH251-128L22 (minus strand)	59807
Macaca mulatta (Mmul)	TCAAGCCTGTCGAGCTTGTG	CH250-541A11 (minus strand)	83781
Callithrix jacchus (Cjac)	TCAAGCCCGTAAAGTATCTGG	CH259-161K20 (minus strand)	52360
Mus musculus (Mmus)	TAGAATAGTTCAGCAGAGACTAC	RP24-208N6 (minus strand)	94231
**T-TSS-II (in *MSY2*):**			
Homo sapiens (Hsap)	CTATGCCAGGGTGCGTTAAGG	RP11-475I1 (plus strand)	54165
Pan troglodytes (Ptro)	CTATGCCAGGGTGCGTTAAGG	CH251-128L22 (minus strand)	60003
Macaca mulatta (Mmul)	CTATGCCAGTGTTCGTTAAGG	CH250-541A11 (minus strand)	83972
Callithrix jacchus (Cjac)	CTCAGGTAGTGTGTGTTAAGG	CH259-161K20 (minus strand)	52543
Mus musculus (Mmus)	CTACCTGAAATGTACAAAGTG	RP24-208N6 (minus strand)	94561
**5´UTR exon-T extension-II<br>(downstream of *MSY2*):**			
Homo sapiens (Hsap)	CTGTGATGCTAAAGCCGTATGC	RP11-475I1 (plus strand)	54263
Pan troglodytes (Ptro)	CTGTGATGCTAAAGCCGTATGC	CH251-128L22 (minus strand)	59906
Macaca mulatta (Mmul)	CTGTGATGCTAAAGCCGTATGC	CH250-541A11 (minus strand)	83880
Callithrix jacchus (Cjac)	CTGTGACGCTAAGGTCGTATGC	CH259-161K20 (minus strand)	52459
Mus musculus (Mmus)	CTGAAACTCCCTACTATCACTTG	RP24-208N6 (minus strand)	94493
**reverse primer: exon 2-3**			
Homo sapiens (Hsap)	CCTTTGCTCGCTGTACTTGC	RP11-475I1 (plus strand)	57788
Pan troglodytes (Ptro)	CCTTTGCTCGCTCTACTTGC	CH251-128L22 (minus strand)	56363
Macaca mulatta (Mmul)	CCTTTGCTCGCTGTACTTGC	CH250-541A11 (minus strand)	80367
Callithrix jacchus (Cjac)	CGTCCTTTGCTTGCTGTACTTGC	CH259-161K20 (minus strand)	49060
Mus musculus (Mmus)	CCTTTGCTCTCTGTATTTCC	RP24-208N6 (minus strand)	91558
