# Supplementary material for: Complex transcriptional control of the AZFa gene DDX3Y in human testis
Source: Int J Androl. 2011 Feb;34(1):84–96. doi: 10.1111/j.1365-2605.2010.01053.x (PMC3039753; doi:10.1111/j.1365-2605.2010.01053.x)
Supplement: Supplementary file 5 [file ijan0034-0084-SD5.pdf]

Table 4, supporting information Rauschendorf et al.

Outcome of the comparative exon-T expression assays in tissues and leukocytes of some primates and human and of mouse as out group. They were aimed to distinguish transcriptional start sites proposed for the human TSS regions in exon-T as described in the main text and in **Table 3 of the supporting information.** Positive RT-PCR reactions are marked by "**+**" and negative RT-PCR reactions are marked by "**–**" sign. Abbreviations used are: Hsap = Homo sapiens, Ptro = Pan troglodytes, Mmul = Macaca mulatta, Cjac = Callithrix jacchus, Mmus = Mus musculus. *DDX3Y* transcripts with 5´UTR extension beyond the TSS-I region into exon-T were only found in the primates´ testis tissue. Interestingly, the testis specific transcripts of Callithrix jacchus seems to start in exon-T in the "T-TSS-I" homologous region. For further discussion see main text.

TSS-region	species	testis	kidney	liver	spleen	leukocytes	water
**TSS-I**	Hsap	+	+	+	+	+	-
	Ptro	+	+	+	+	+	-
	Mmul	+	+	+	+	-	-
	Cjac	+	+	+	+	+	-
	Mmus	+	+	+	+	+	-
**5´UTR exon-T extension-I**	Hsap	+	-	-	-	-	-
	Ptro	+	-	-	-	-	-
	Mmul	+	-	-	-	-	-
	Cjac	+	-	-	-	-	-
	Mmus	-	-	-	-	-	-
**5´UTR exon-T extension-II**	Hsap	+	-	-	-	-	-
	Ptro	+	-	-	-	-	-
	Mmul	+	-	-	-	-	-
	Cjac	-	-	-	-	-	-
	Mmus	-	-	-	-	-	-
**T-TSS in *MSY2***	Hsap	+	-	-	-	-	-
	Ptro	+	-	-	-	-	-
	Mmul	+	-	-	-	-	-
	Cjac	-	-	-	-	-	-
	Mmus	-	-	-	-	-	-
